# Cognitive foundations of organizational learning: re-introducing the distinction between declarative and non-declarative knowledge

**DOI:** 10.3389/fpsyg.2015.01489

**Published:** 2015-09-30

**Authors:** Barbara Kump, Johannes Moskaliuk, Ulrike Cress, Joachim Kimmerle

**Affiliations:** ^1^Knowledge Construction Lab, Knowledge Media Research Center, TuebingenGermany; ^2^Department of Human Resources and Organisation, University of Applied Sciences for Management and Communication, ViennaAustria; ^3^Department of Applied Cognitive Psychology and Media Psychology, University of TuebingenTuebingen, Germany

**Keywords:** organizational learning, declarative knowledge, non-declarative knowledge, co-evolution, organizational routines, skills, habits, organizational practice

## Abstract

Contemporary research into socio-cognitive foundations of organizational learning tends to disregard the distinction between declarative and non-declarative knowledge. By reviewing the literature from organizational learning research and cognitive psychology we explain that this distinction is crucial. We describe the foundations of organizational learning by referring to models that consider the interplay between individual and collective knowledge-related processes in organizations. We highlight the existence of a research gap resulting from the finding that these approaches have widely neglected the existence of different types of knowledge. We then elaborate on characteristics of declarative and non-declarative knowledge in general, consider organizations as structures of distributed cognition, and discuss the relationship between organizational knowledge and practice. Subsequently, we examine the role of declarative and non-declarative knowledge in the context of organizational learning. Here, we analyze (1) the cognitive and social mechanisms underlying the development of declarative and non-declarative knowledge within structures of distributed cognition; and (2) the relationship between alterations in declarative and non-declarative types of knowledge on the one hand and changes in organizational practice on the other. Concluding, we discuss implications of our analysis for organizational learning research. We explain how our integrative perspective may offer starting points for a refined understanding of the sub-processes involved in organizational learning and unlearning and may support a better understanding of practical problems related to organizational learning and change.

## Introduction

Organizational learning is based on continuous alterations in an organization’s repertoire of knowledge, which broaden its potential range of organizational practice; such changes in practice, in turn, may lead to the development of new knowledge (Fiol and Lyles, 1985). Understanding how organizational learning takes place within organizations, therefore, requires an understanding of (1) how knowledge is being developed, and (2) how the development of knowledge and practice mutually influence each other. These questions are at the core of organizational learning research.

Since the publication of seminal work, for example, by Fiol and Lyles (1985) and Levitt and March (1988), many different disciplines have explored the foundations of organizational learning from various perspectives (Nicolini and Meznar, 1995; Easterby-Smith, 1997; Popper and Lipshitz, 2000; Schulz, 2002; Bapuji and Crossan, 2004; Argote and Miron-Spektor, 2011; Argote, 2013). Many of the existing prominent models posit that organizational learning is based on the interplay of cognitive (individual) and social mechanisms (March, 1991; Kim, 1993; Nonaka and Takeuchi, 1995; Spender, 1996; Crossan et al., 1999). In these models, however, the underlying cognitive and social processes and their interrelations have not been sufficiently specified.

More recent socio-cognitive approaches have developed these perspectives further. These approaches view organizational learning as the outcome of reciprocal interactions among cognitive and social processes that are embedded in organizational structures and cultures (Akgün et al., 2003; Elkjaer, 2004; Casey, 2005; Antonacopoulou and Chiva, 2007; Brusoni and Rosenkranz, 2014). Organizational knowledge is conceived to be actively produced and co-constructed by individuals with differently embedded beliefs, values, and practices. These approaches have explained many of the complex phenomena involved in organizational learning. Nevertheless, there is an important white spot in this socio-cognitive stream of research. While older models of organizational learning (Cohen, 1991; Kogut and Zander, 1992; Kim, 1993; Nonaka and Takeuchi, 1995) were aware of the distinction between declarative and non-declarative knowledge, more recent approaches refer broadly to “knowledge” and do not distinguish between these concepts. This is problematic as neuropsychological research has identified substantial structural and functional differences in declarative and non-declarative memory systems (Anderson, 1996; Squire, 2004; Sun, 2012; Conti-Ramsden et al., 2015). These differences in memory systems are responsible for how the cognitive foundations of organizational learning processes are built. They shape the individual and collective, that is, cognitive and social mechanisms involved in organizational learning. It is the aim of the present article to further develop the current understanding of organizational learning by re-introducing the distinction between declarative and non-declarative knowledge.

We will discuss the consequences of this distinction with regard to both the development of knowledge and changes in organizational practice. We will synthesize existing conceptual contributions and empirical findings from organizational learning research and cognitive psychology to explain that (1) different mechanisms underlie the development of declarative and non-declarative knowledge within organizations, and that (2) declarative and non-declarative knowledge each contribute in a different way to changes in organizational practice. This distinction between declarative and non-declarative knowledge may not only enhance theory in continuous organizational learning, but may also enable a better understanding of practical problems.

In the following sections, we first provide a brief overview of research into foundations of organizational learning: We introduce the distinction between declarative and non-declarative knowledge, characterize organizations as structures of distributed cognition, and describe the relationship of knowledge and practice within organizations. Secondly, we build on existing research to elaborate on how each of the two types of knowledge are developed and how this development is related to alterations in organizational practice. In the concluding section, we discuss implications of this analysis for future research.

## Foundations of Organizational Learning

Two of the most seminal models that have established the groundwork for research into the foundations of organizational learning are Nonaka and Takeuchi’s (1995) SECI model (Teece, 2013), and Crossan et al.’s (1999) 4I framework. Both models aim to explain the concrete and complex, individual and collective, and cognitive and social mechanisms involved in organizational learning. The SECI model describes how knowledge is co-created by individuals within organizations through continuous verbal and non-verbal communication. According to this model, individuals are introduced into a social system through socialization, in the course of which they learn from each other mainly through co-experience. If individuals externalize their knowledge to others (i.e., express it verbally), knowledge of multiple individuals can be combined, thereby leading to new insights. These new insights are then again internalized (i.e., learned) by the individuals involved.

The 4I framework has a slightly different focus than the SECI model. Its purpose is to explain the different phases within an organizational learning process by describing how knowledge transcends from the individual to the team and organizational levels. According to the 4I framework, organizational learning involves four phases, intuiting, interpreting, integrating, and institutionalizing. In the intuiting phase, knowledge is created based on experience at the individual level. In the interpreting stage, the individual links the newly generated knowledge with existing knowledge, before that knowledge is shared with a group of colleagues in the integrating phase. In the institutionalizing phase, routinized actions are developed, tasks defined, actions specified, and organizational mechanisms put into place.

Both the SECI model and the 4I framework provide starting points for understanding how individual and collective knowledge co-evolve within organizations through the interplay of different cognitive mechanisms and communication processes. While the co-creation of individual and collective knowledge is the explicit focus of the SECI model, the 4I framework also describes how knowledge is transferred from the individual to the collective through cognitive mechanisms (intuiting and interpreting) and social processes (integrating and institutionalizing). In addition, the relationship of knowledge and practice plays an important role in both models: In the SECI model, socialization is based on knowledge sharing during collective practice. In the 4I framework, the organizational learning process is not finished until the newly generated knowledge is institutionalized as common practice. Nevertheless, both models tend to remain vague about the concrete, underlying cognitive and social mechanisms, leaving important questions unanswered, such as “How exactly do individuals create new knowledge?”, “How do different individuals combine their ideas?”, or “How can processes, once institutionalized, be revised?”

Expanding on the SECI model, the 4I framework, and other concepts, more recent socio-cognitive approaches have analyzed how individuals co-create knowledge within organizations, and how the development of knowledge is related to the development of practice (Akgün et al., 2003; Berends et al., 2003; Elkjaer, 2004). These approaches have integrated important psychological concepts such as emotions, intelligence, or improvisation (Akgün et al., 2003), have shown that organizational practices are actively structured (Berends et al., 2003), and have developed insights into how organizations and individuals mutually shape each other (Elkjaer, 2004). However, those same approaches have widely neglected the existence of different types of knowledge. The need for such a distinction, especially between declarative and non-declarative types of knowledge, has clearly been highlighted both in previous research in the field of cognitive psychology (Anderson, 1996) and in earlier models of organizational learning (Cohen, 1991; Kogut and Zander, 1992; Kim, 1993) and is also implied in Nonaka and Takeuchi’s (1995) SECI model.

In the present article, we will elaborate on characteristics of declarative and non-declarative knowledge in the context of organizational learning and demonstrate that these knowledge types strongly differ with respect to (1) how they are developed within organizations, and (2) how they are related to organizational practice. In order to lay the groundwork for our overall conceptualization, we will clarify the differences between declarative and non-declarative knowledge types, analyze organizations as structures of distributed cognition where cognitive and social processes mutually influence each other, and describe the general relationship between knowledge and practice within organizations.

### Declarative and Non-declarative Knowledge

Research in cognitive psychology and neuro-psychology has identified substantial structural and functional differences between declarative and non-declarative memory (Squire, 2004; Sutton et al., 2010; Sun, 2012). Declarative memory refers to the capacity for the recollection of facts and events, which allows for comparing and contrasting remembered material. Examples of declarative memory are a pharmacist’s retention of knowledge about different types of drugs and their effects (facts), or a consultant’s retention of knowledge of different cases she has been working on (events). Non-declarative memory refers to several additional instances of memory that are expressed through performance rather than through recollection. Non-declarative memory is the store of non-declarative knowledge, such as skills and habits (Squire, 2004). Examples of skills are motor skills, like a sculptor’s ability to carve stone, or cognitive skills, such as an interpreter’s ability to simultaneously translate spoken words into a different language. Examples of habits include a farmer’s process of feeding cattle every morning or an office worker’s particular way of starting the work day by turning on his computer and checking e-mails.

Human memory has three main tasks (Sutton et al., 2010). First, incoming information must be processed and “encoded”; second, information must be retained and “stored” in some way; third, information must be accessed and “retrieved”. Declarative and non-declarative memories operate according to different principles with regard to these three stages (Squire, 2004; Ferdinand et al., 2010). In the case of declarative memory, an important operational principle is the ability to detect, encode, and store what is general and what is unique about a single entity. In other words, declarative memory is about making distinctions which lead to the development of increasingly abstract representations of concepts. Non-declarative memory, in contrast, is procedural (i.e., knowing how to do things) and embodied; the key mechanism for building non-declarative knowledge, such as habits and skills, is the repetition of the same procedures.

Sometimes, this distinction between declarative and non-declarative knowledge is equated or at least closely related (Argote and Miron-Spektor, 2011) with the distinction between tacit and explicit knowledge as introduced by Polanyi (1967, 1975). Tacit knowledge is characterized by the fact that it is difficult to express verbally. That is, someone may know something, but is not able to transfer this knowledge to someone else in words. Explicit knowledge, in contrast, is verbally codified and may thus be expressed verbally, which makes it easier to transmit to other people. Considering the properties of declarative and non-declarative memories described above, it can be concluded that knowledge which is stored in non-declarative memories tends to be tacit (hard to express verbally, “embodied”), whereas knowledge which is stored in declarative memories is basically explicable. However, Polanyi (1967) stressed that explicit knowledge always has a tacit basis and people may be able to express some types of (previously) tacit knowledge linguistically if they focus their attention upon it (Tsoukas, 1996). In other words, tacit knowledge is a necessary component of all knowledge and to a large extent tacitness is more a matter of focus and attention than a property of a certain “piece of knowledge”. As a consequence, the distinction between tacit and implicit knowledge is not identical with the distinction between declarative and non-declarative knowledge types. The distinction between declarative and non-declarative knowledge is based on structural and functional differences and is, therefore, more relevant and guiding to organizational learning research than the distinction between tacit and explicit knowledge.

### Organizations as Structures of Distributed Cognition

According to socio-cognitive perspectives, organizational learning is based on modifications in individual knowledge, that is, on individual cognitive processes. Therefore, comprehension of these cognitive processes is relevant to understanding organizational learning mechanisms. Individual phenomena alone, however, cannot explain complex organizational phenomena, which is what organizational learning involves. In order to understand how knowledge is created within organizations, it is necessary to understand how cognitive and social processes are related (Foss et al., 2010; Hodgson, 2012). Explanations of organizational phenomena must be grounded in explanations that include both individual and social relations.

Multiple concepts have been presented that aim to describe how knowledge of different individuals is being integrated within organizations (Tell, 2011). In this article, we follow the socio-cognitive tradition that regards organizations as social structures of distributed cognition, where individual knowledge is continuously being exploited and further developed through the communication, integration, and combination of individually held declarative and non-declarative knowledge (Tsoukas, 1996; Thompson et al., 1999; see also Hutchins, 1995a,b; Salomon, 1997; Baber et al., 2014). This idea of distributed knowledge that exists within the organization and that can be combined to successfully accomplish organizational tasks has been incorporated into a number of theories, such as in the communities of practice model (Wenger, 1998; Wenger and Snyder, 2000; Brown and Duguid, 2001; Kimmerle et al., 2013), or in the concept of transactive memory systems (Wegner, 1986; Lewis and Herndon, 2011; Hecker, 2012; Hewitt and Roberts, 2015).

In such structures of distributed cognition, complementary knowledge of different individuals is accessed through the social networks and relationships of people within the organization. In aviation, for example, an air-traffic controller and a pilot need to combine their complementary knowledge of the current situation (the air-traffic controller has knowledge of the situation in the airspace and at the airport, and the pilot has knowledge of the situation on the plane) and closely collaborate to make sure that a plane can land safely. In addition to knowledge that exists in individual cognitive systems, collective knowledge within organizations may be embedded in artifacts (e.g., documents, wiki texts, and databases) which can be accessed by the organization’s members to achieve their goals (Riss et al., 2007; Hecker, 2012). In order for the structure of distributed cognition to be efficient, however, it is essential that there is not only complementary knowledge but also “common ground” (Clark and Brennan, 1991), that is, some knowledge that is shared among group members. In order to collaborate successfully, both the pilot and the air traffic controller in the example above need shared knowledge on flight regulations and on basic meteorological principles.

In line with these perspectives, we regard knowledge within organizations as distributed (complementary or shared) knowledge that exists at different locations (in the cognitive systems of individuals as well as in artifacts) and that is continuously being further developed through different forms of communication. Because distributed knowledge may be declarative or non-declarative and may appear in different shapes (van den Berg, 2013), communication here is broadly defined as the process by which individuals interact and influence each other (Craig, 1999). This definition includes oral communication, communication via shared artifacts (e.g., documents and devices), and non-verbal forms of communication, such as watching another person carrying out an activity.

### Organizational Knowledge and Practice

Another prerequisite for developing further the foundations of organizational learning is to understand the relationship between organizational knowledge and practice. In general, knowledge and practice are strongly interrelated in their development, as knowledge is both sustained in practice and manifests itself through practice (Berends et al., 2003; Elkjaer, 2004; Nicolini, 2011). One important aspect of organizational learning is the process of generating modifications in knowledge through practice. Conversely, the acquisition of new knowledge may lead to the improvement of existing practice (e.g., increased efficiency) or it may lead to the development of new practice through better knowledge (Crossan et al., 1999; Feldman, 2000). A modification in organizational knowledge may enable organizational unlearning (intended modification of practice) or result in unintended organizational forgetting (Tsang and Zahra, 2008). Finally, organizational knowledge may not be exploited in practice at all, but only exist as latent potential for behavior (Hargadon and Fanelli, 2002).

There is not a one-to-one correspondence between knowledge and practice: Knowledge does not always result in respective practice and changes in practice may be observed in organizations that cannot be directly traced back to changes in particular knowledge structures (Fiol and Lyles, 1985; Huber, 1991; Crossan et al., 1999).

## Declarative and Non-declarative Knowledge in Organizational Learning

In order to further advance research into foundations of organizational learning based on the distinction between declarative and non-declarative knowledge, an analysis is needed which leads to a refined conceptualization of

(1) the cognitive and social mechanisms underlying the development of declarative and non-declarative knowledge within structures of distributed cognition, and
(2) the relationship between alterations in declarative and non-declarative types of knowledge, and alterations in organizational practice.

Therefore, in the following sections, we first integrate existing research from multiple disciplines to adopt a co-evolution perspective which enables us to consider in detail the cognitive and social mechanisms involved in the development of declarative and non-declarative knowledge within organizations. Then, we synthesize findings from existing research to describe the different ways in which changes in declarative and non-declarative knowledge are related to changes in practice within organizations.

### The Co-evolution of Individual and Collective Knowledge

As outlined above, existing socio-cognitive approaches do indeed analyze how individuals co-create knowledge within organizations (Akgün et al., 2003; Berends et al., 2003; Elkjaer, 2004), but they do not elaborate on differences between the declarative and non-declarative types of knowledge in these collaborative learning processes. One model that provides a first step in this direction is the co-evolution model by Kimmerle et al., 2010a; see also Cress and Kimmerle, 2008; Oeberst et al., 2014; Kimmerle et al., 2015).

Kimmerle et al. (2010a) investigated individual and collective mechanisms involved in the co-evolution of organizational knowledge through shared digital artifacts such as wiki articles or social tagging systems. Their model integrates Luhmann’s (1995) theory of social systems and Piaget’s (1977) theory of cognitive development and considers individual learning and collaborative knowledge building (Hewitt and Scardamalia, 1998; Scardamalia, 2002; Scardamalia and Bereiter, 2006) as two interrelated co-evolutionary processes: When people co-construct knowledge through shared digital artifacts, the learning processes of different individuals mutually influence each other (Halatchliyski et al., 2014). Kimmerle et al. (2010a) introduced a distinction between declarative and non-declarative knowledge and briefly touched on how knowledge co-evolution takes place for both of these knowledge types within digital artifacts. In our analysis, we will advance this approach and enrich it with further findings on the co-evolution of declarative and non-declarative knowledge within organizations.

#### The Co-evolution of Individual and Collective Declarative Knowledge

Within structures where cognition is distributed among many individuals, as in a corporate organization, the combination of pieces of declarative knowledge held by different individuals enables the creation of new collective declarative knowledge and ideas (Gibson, 2001). This knowledge creation is based on different cognitive and social mechanisms.

Schema theory and related concepts of mental representations pervade contemporary research on cognition in organizations (Hodgkinson and Healey, 2008); that is, individual declarative knowledge is assumed to be stored in the form of cognitive schemas (Bartlett, 1932; Greeno, 1980). A schema is a mental model which contains an individual’s conjectures about the world (Axelrod, 1973) and is regarded as a cognitive representation of previous experiences with similar features (Gick and Holyoak, 1983). A schema allows an individual to infer information that is not part of a current experience, based on existing knowledge about previous, similar experiences (Greeno, 1980). As a mental data structure, the schema guides the perception and processing of stimuli from the environment, and all new knowledge is interpreted against the backdrop of the existing schema. For instance, a chef who has a cognitive schema of a “restaurant kitchen” will be able to handle the oven and know which ingredients are usually available in the storeroom and the freezer, respectively, even when he enters the kitchen of a particular restaurant for the first time.

Based on neuropsychological studies, Ghosh and Gilboa (2014) identified four necessary features of schemas. First, schemas have an associative network structure; that is, they comprise of units and their relationships. Second, schemas are being developed on the basis of multiple experiences; they represent the similarities and commonalities across events. Third, schemas lack of unit detail, which follows from that they are based on multiple experiences and episodes. Fourth, schemas are adaptable; they are constantly developing, based on incoming new information.

This adaptability of cognitive schemas is at the core of how learning takes place in the declarative memory system. Adaptation of a cognitive schema is triggered by an incongruity between information encountered in the external world and the prior knowledge of a person triggers a cognitive conflict; this cognitive conflict leads to modifications in individual cognitive structures through processes of accommodation or assimilation (Piaget, 1977; Ghosh and Gilboa, 2014). Assimilation occurs when new information is added to the existing prior knowledge without modification of the existing schemas. In an accommodation process, in contrast, existing schemas have to be changed in order for new information to fit. While assimilation means accumulating additional information into the existing structures, accommodation means that cognitive systems typically become more complex or more sophisticated (Rumelhart and Norman, 1978; Moskaliuk et al., 2009, 2011). For example, an engineer in an automotive company may learn about an improved (more efficient) component for a fuel-injection system in a combustion engine. This new knowledge may be assimilated into her existing schema of “how engines work”, because the basic principle of the engine’s functionality remains unchanged from her point of view. However, if an engineer for combustion engines acquires knowledge about the components of an electric motor, the existing schema of “how engines work” may become more complex in its accommodation of the new information.

Individuals change their cognitive schemas (i.e., they learn) as a result of their social interaction with their environment (Zerubavel, 1997), especially through verbal communication with other individuals. If individuals verbally communicate with each other within structures of collectively distributed cognition, it is likely that they are confronted with information that is incongruent with their existing prior knowledge. As a consequence, they may have to adapt their existing schemas in order to resolve the cognitive conflict. To resolve the cognitive conflict, individuals may either assimilate new information into any prior knowledge or modify some existing knowledge to accommodate new insights. In oral conversations, such accommodation and assimilation processes are difficult to observe. However, these processes have been demonstrated in situations where knowledge was being co-created via shared digital artifacts (Kimmerle et al., 2010b).

On the basis of these findings, the following mechanisms can be assumed to be at play in the development of individual declarative knowledge within a co-evolution cycle: Through verbal communication (in meetings, e-mails, and documents), individuals may externalize specific parts of their declarative knowledge. This externalized knowledge may be internalized by other people. During these internalization processes, individuals use their existing cognitive schemas to interpret novel information. An incongruity between existing knowledge and new information may lead to a cognitive conflict. This cognitive conflict then triggers adaptations of the cognitive schema of the individual that experiences the conflict, either through assimilation of new information into the existing schema or accommodation of the schema.

As a consequence, repeated verbal communication within a system of distributed cognition leads to the recursive adjustment of individually held schemas within organizations, which then become more and more alike over time (Zerubavel, 1997). That is, individuals develop shared knowledge in the sense that parts of their cognitive structures become similar over time. Besides creating shared knowledge, individuals will build complementary declarative knowledge as a consequence of task specialization, based on a division of knowledge responsibilities (Lewis and Herndon, 2011; Hecker, 2012).

**FIGURE 1:**
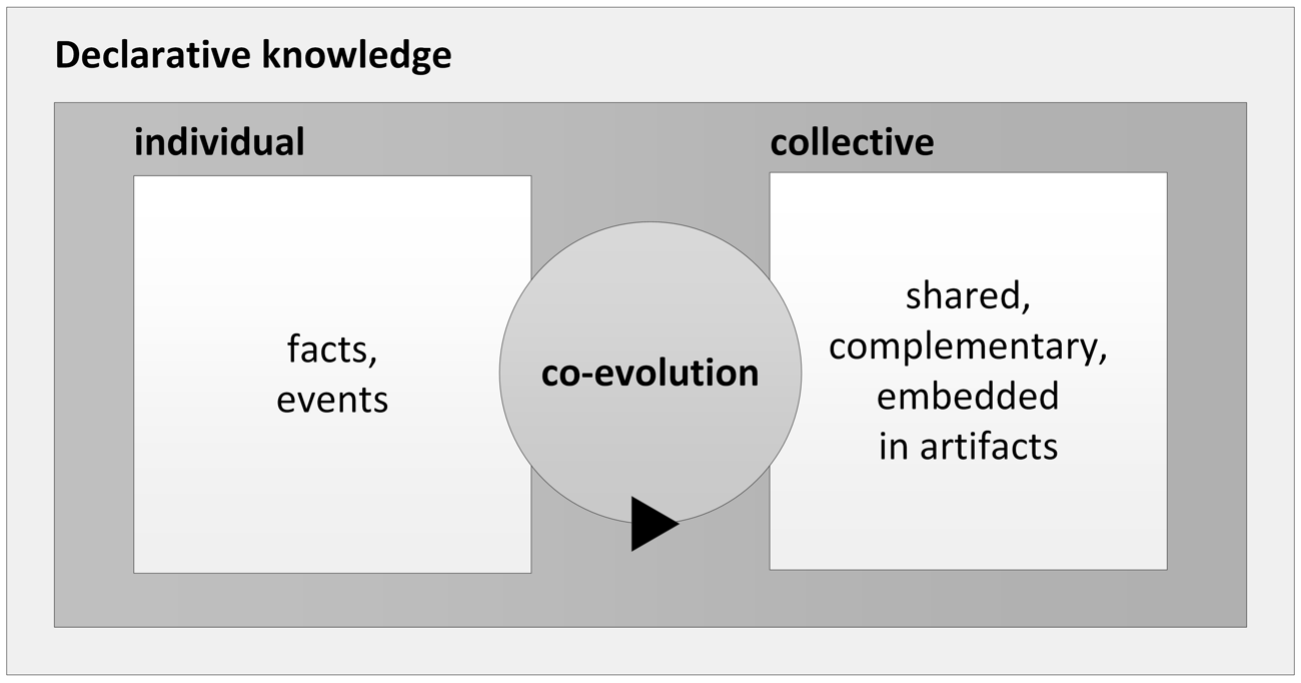
**The co-evolution of individual and collective declarative knowledge**.

In summary, we conclude from previous research that the co-evolution of individual and collective declarative knowledge within organizations happens mainly through individuals’ sharing knowledge with others through verbal communication, including communication via shared artifacts that are verbally coded. In this way, the knowledge of each individual expands in assimilation and accommodation processes. Externalization and internalization of knowledge lead to the recursive adjustment of individually held schemas within organizations and to the formation of collective declarative knowledge (**Figure 1**).

#### The Co-evolution of Individual and Collective Non-declarative Knowledge

With regard to the co-evolution of individual and collective non-declarative knowledge, the questions must be answered as to how skills and habits are developed by individuals and how they are related with collective non-declarative knowledge within structures of distributed cognition.

Cognitive psychology has found that skills such as the psychomotor skill of writing on a typewriter or the cognitive skill of calculating the square-root of a two-digit number are acquired through exercise, that is, through repetition of the same activities (Anderson, 1982; VanLehn, 1996; Sun et al., 2001; Wiggins et al., 2014). Skill acquisition can be understood as skill composition, that is, combining component skills in novel ways or adapting already-known component skills to enable the performance of new tasks (Salvucci, 2013).

Habits, as another form of non-declarative knowledge, denote an individual’s customary ways of behaving (Ouellette and Wood, 1998; Adriaanse et al., 2014). They are controllable only to a limited extent, executed mostly without awareness, and tend to be efficient (Verplanken and Orbell, 2003). For example, an experienced mechanic will employ a very similar procedure each and every time he changes the wheels of a car. Likewise, a head physician will choose the same path every morning on the ward round, ask similar questions and check the same medical indicators.

Habits are intentional and goal-directed in their origins (Ouellette and Wood, 1998), meaning that at the beginning of the formation of a habit, a certain behavior (e.g., taking the bus) was executed intentionally in order to achieve a particular goal (e.g., getting to work). Like skills, habits are developed through repetition (Ouellette and Wood, 1998; Wood and Neal, 2007). Danner et al. (2007, p. 1369) argue that “habit formation occurs when the same means is repeatedly and consistently retrieved for the same goal because it promotes an automatic search for and access to these means in memory”. That is, by satisfactory repetition, a certain practice may become habitual (Verplanken and Orbell, 2003). When a habit has been formed, a specific response is spontaneously triggered by specific cues in the environment (Wood and Neal, 2007; Neal et al., 2012), such as the wheel requiring changing together with the car in a specific position in the mechanic example. Environmental cues are essential for the habit-formation process.

Collective (distributed) non-declarative knowledge has been the focus of a large body of research into formation of routine: “the routine of a group can be viewed as the concatenation of such procedurally stored actions, each primed by and priming the actions of others” (Cohen and Bacdayan, 1994, p. 557). The knowledge that underlies the parts of the routine carried out by the individual actors is often not articulated.

Routines appear prominently and persistently in the description of organizational action and organizational learning (Feldman, 2000; Becker, 2004; Cohen, 2007; Pentland et al., 2011; Rerup and Feldman, 2011). Research into routine formation has shown that the development of non-declarative (individual) knowledge plays an important role in the formation of organizational routines (Cohen and Bacdayan, 1994; Cohen, 2012; Cohen et al., 2014). At the outset of the development of organizational routines, individuals form goals and intentions to act and perform a certain behavior to achieve these goals (Ouellette and Wood, 1998; Bapuji et al., 2012). The feedback about the behavior (success or failure) comes along with positive or negative reinforcement, which may increase or decrease the likelihood of its repetition. If successful, the repetition of behavior may lead to the formation of individual habits and skills after some trials.

During the execution of a routine, individuals may communicate verbally and non-verbally. Routine formation is more likely if at the outset the intention of the routine is clearly communicated and unambiguously conveyed by “intermediaries” (Bapuji et al., 2012). Bapuji et al. (2012, p. 1589) define an intermediary as “an entity that moves between actors performing a task and transmits the intentions of one actor to another”. Such intermediaries can take many forms, such as computer software, contracts, or technological artifacts.

**FIGURE 2:**
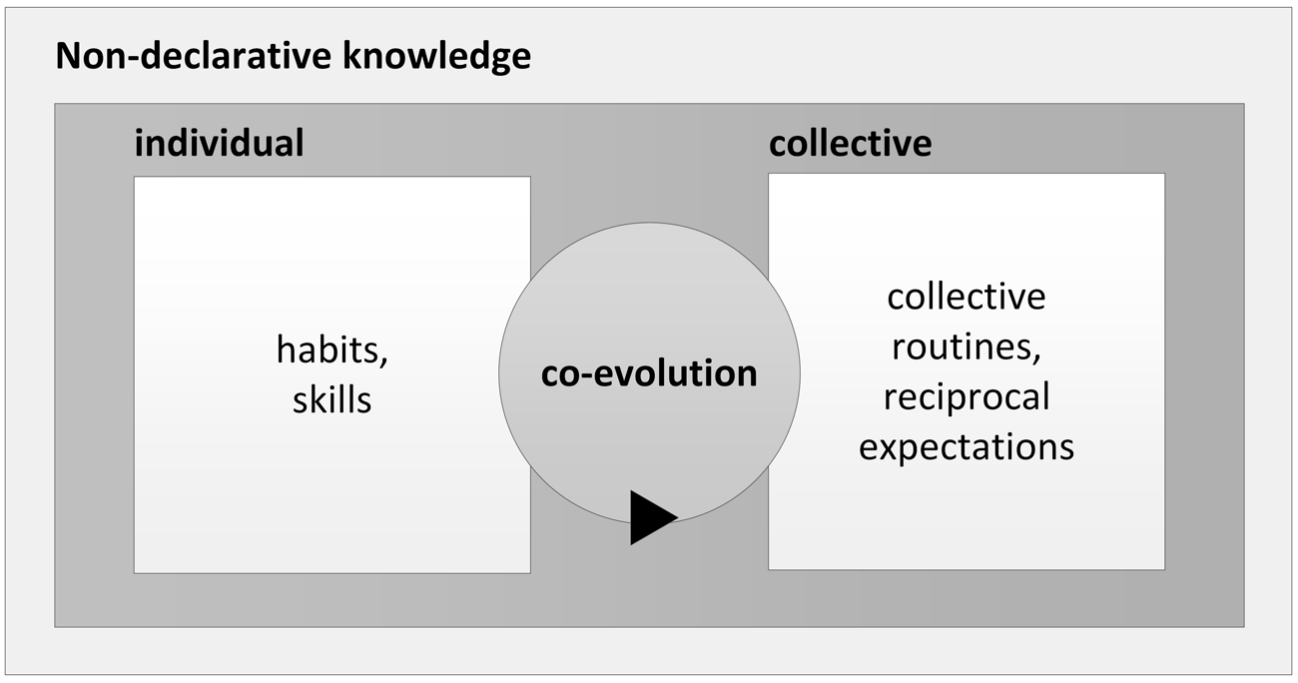
**The co-evolution of individual and collective non-declarative knowledge**.

In collective practice, each individual triggers and carries out some form of behavior to achieve a goal. Once a routine has been established, the non-verbal behavior of one individual, together with an intermediary, serves as behavioral “cue” to other individuals who, in turn, trigger new behavior of other individuals (Cohen and Bacdayan, 1994; Bapuji et al., 2012). Through repeated co-experience, the intentions of the social interactions no longer need to be verbalized and turn into reciprocal expectations (Thorngate, 1976; Howard-Grenville, 2005). As an example for reciprocal expectations in organizational learning, consider an experienced team of firefighters trying to extinguish a fire: When a fireman starts unwinding the fire hose and runs to the source of the fire, he “knows” that a colleague in the fire truck will turn on the water in due course, because the colleague has done so in many previous instances. In summary, existing research suggests that individual non-declarative knowledge within organizations develops through repeated individual practice. The co-evolution of individual and collective non-declarative knowledge takes place on the basis of repeated collective practice and mutual reinforcement of habituated behavior which, over time, may lead to the formation of reciprocal expectations and converge into organizational routines (**Figure 2**).

### The Relationship between Declarative and Non-declarative Knowledge and Changes in Organizational Practice

**FIGURE 3:**
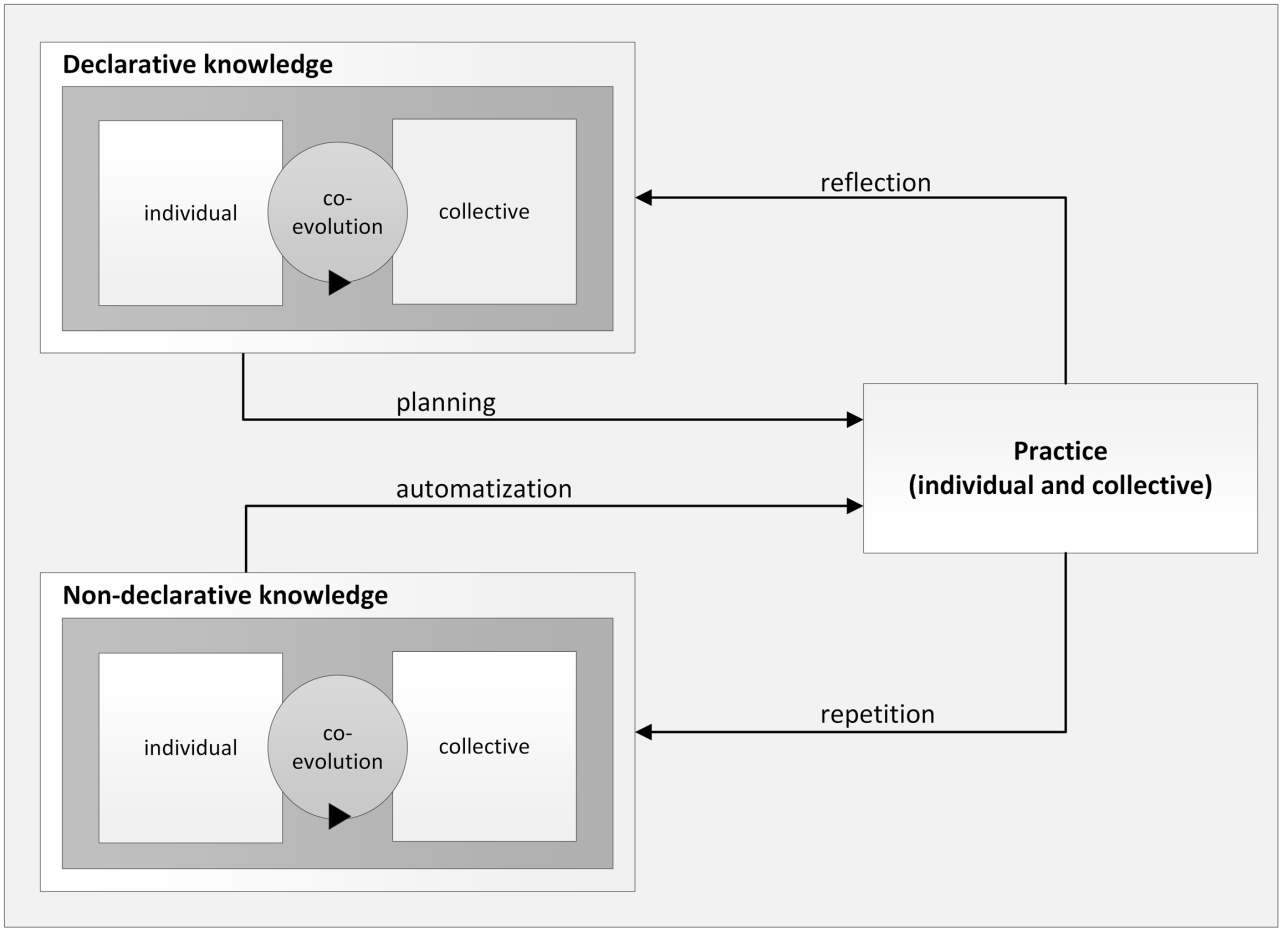
**The interplay of declarative and non-declarative types of knowledge with practice**.

So far, we have described the different mechanisms underlying the co-evolution of declarative and non-declarative knowledge within organizations. We have elaborated on the relationship between knowledge and practice within organizations in general terms. The distinction between declarative and non-declarative knowledge now allows us to refine these general conclusions about the relationship between knowledge and practice. In the following, we will elaborate on differences between declarative and non-declarative knowledge with regard to the modification of practice. For an overview in this context, **Figure 3** summarizes our main considerations.

#### Declarative Knowledge and Changes in Organizational Practice

Organizational practice is based and dependent upon both individual and collective declarative knowledge (Fiol and Lyles, 1985): Individuals rely on their existing factual and episodic knowledge to perform organizational tasks. More complex tasks may involve multiple individuals and require a combination of the declarative knowledge of those individuals.

Reflection upon current (individual or collective) practice may lead to the formation of new declarative knowledge (Dewey, 1933; Tsoukas and Vladimirou, 2001; Høyrup, 2004; Knipfer et al., 2013). In a given situation, an individual within an organization may make a judgment concerning a situation at hand and begin to search (consciously or unconsciously) for an appropriate response (Tsoukas and Vladimirou, 2001). This judgment may prove adequate if the expected results occur, or inadequate if the expected results do not occur. If the expected results do not occur the individual may experience a discrepancy between the expected and the actual outcomes (Knipfer et al., 2013); this discrepancy may result in cognitive discomfort. Such cognitive discomfort is assumed to trigger a reflection process, which may result in the creation of new declarative knowledge (through modification of existing schemas). Reflection may take place individually or collectively (Renner et al., 2014). If the outcomes of the reflective process are verbalized to other individuals, this may trigger co-evolution cycles of declarative knowledge.

Alterations in declarative knowledge may enable individuals to articulate and achieve new individual and collective goals and come up with novel strategies to achieve these goals. In this way, new declarative knowledge has an impact on practice, in that new intentions may be formed and new activities planned and implemented. For example, an individual may realize that an existing practice could be improved and may intentionally modify his or her own existing (habituated) practice through formulating new goals or thinking of new, more efficient ways to carry out previously automatized tasks. Such alterations in declarative knowledge may not only lead to alterations in individual practice, but may also result in the intention to modify existing collective routinized behavior. A similar mechanism was described by Gavetti and Levinthal (2000), who modeled the process of how cognition-based decisions enable the accumulation of experiences through modified practice. Whether or not these intentional changes of organizational routines actually lead to changes in organizational practice may depend on different factors, such as the alignment of the intended change in performance with the original purpose of the routine (Feldman, 2003). When repeated, the new goal-oriented practices may lead to the formation of new individual habits (Ouellette and Wood, 1998; Wood and Neal, 2007; Neal et al., 2012) or collective routines (Cohen and Bacdayan, 1994; Bapuji et al., 2012).

#### Non-declarative Knowledge and Changes in Organizational Practice

An even closer link exists between the formation of non-declarative forms of knowledge and alterations in organizational practice. Non-declarative knowledge, as we have explained above, is built through repetition of practice. Both skills and habits can be acquired only through practice and they can only manifest themselves in practice. Repetition leads to the formation of individual skills and habits and—through co-evolution cycles—to collective routines which manifest in automatized practice.

Clearly, not all practice within organizations is automatized and based on habit, but through by-passing cogitation habit formation frees individuals to use their finite information-processing capacity for other kinds of problem solving (Thorngate, 1976; Argyris, 1977; Wood and Neal, 2007). This gain in efficiency through automatization appears to be particularly relevant under conditions of heavy load, such as exhaustion, time pressure, distraction, or information overload (Verplanken and Orbell, 2003). Successful outcomes (through better skills and more efficient procedures) increase the likelihood of the pertinent behavior to occur. An equivalent mechanism is involved in collective reinforcement learning from the past, which also results in the formation of collective routines (Feldman, 2000; Gavetti and Levinthal, 2000; Pentland et al., 2010, 2011). Therefore, within organizations—where there is often a demand to deal quickly and efficiently with rather familiar situations—there is a tendency toward reduction of complexity and behavior is likely to become routinized.

Combining these observations, we conclude that existing non-declarative knowledge, both at the individual (skills and habits) and the collective levels (routines and reciprocal expectations), enables organizations to employ practices and attain goals in an automatic and efficient way, with predictable outcomes and products. The skills of the individuals involved in the routine are thereby improved, automatization of individual and collective behavior is reinforced, and reciprocal expectations develop. Through repetition, non-declarative knowledge is formed within organizations, which leads to increasing stabilization of organizational practice (in the form of habits and routines).

While there is a tendency for habituation and routine formation (through co-evolution cycles of non-declarative knowledge), routinized practice does not necessarily have to be rigid. On the contrary, routines have been found to be rather flexible entities (Feldman, 2000; Levinthal and Rerup, 2006; Cohen, 2007; Pentland et al., 2010; Miller et al., 2012; Turner and Fern, 2012): The basic structure of a routine can remain relatively stable, while the actual manifestation of practice can display substantial variety, as routines are being enacted anew in each execution. Different forms of variation in practice (Levinthal and Marino, 2015) may lead to the development of new skills and modified habits. These newly generated skills and habits may in turn shape the execution of the routine the next time around (Levinthal and Rerup, 2006; Cohen, 2007; Miller et al., 2012). Through improved skills, individuals may show new behaviors or apply different sub-procedures, which then slowly change the routine as a whole.

## Discussion and Conclusion

The intention of this article was to contribute to the further development of the concrete individual and collective processes underlying organizational learning by bringing the distinction of declarative and non-declarative knowledge back into the debate. Starting from the distinction of these two forms of knowledge, we first synthesized findings from organizational learning research and cognitive psychology to show that co-evolution of individual and collective knowledge differs between declarative and non-declarative knowledge: on the organizational level, collective declarative knowledge (shared knowledge, complementary knowledge, and knowledge documented in artifacts) evolves mainly through verbal communication. Collective non-declarative knowledge (collective routines and reciprocal expectations) evolves mainly through repeated practice. Verbal communication may incite cognitive conflicts in individual cognitive schemas, which in turn trigger the development of individual declarative knowledge through assimilation and accommodation processes. Non-verbal forms of communication, such as collaboration and co-experience in common tasks, may result in the formation of individual habits and skills, that is, individual non-declarative knowledge. In making this distinction between declarative and non-declarative knowledge, we include an analysis of individual level cognitive processes (assimilation, accommodation, and formation of habits and skills) in organizational learning, which have commonly been ignored in contemporary socio-cognitive approaches.

As a second contribution, we have described the different effects of declarative and non-declarative knowledge on the formation of organizational practice. This integrative view could serve to further enhance recent theoretical considerations. For instance, it may add to the debate on “organizational unlearning”, which has been described as the “discarding of old routines to make way for new ones, if any” (Tsang and Zahra, 2008, p. 1437; italics removed by the authors). Tsang and Zahra (2008) explicitly state that organizational unlearning incorporates behavioral and cognitive dimensions. They explain that unreflective, habitual actions may be intentionally changed or discarded as a consequence of “cognitive activities”. Applying our integrative view allows us to be more specific about this mechanism of intentional organizational unlearning: for example, a certain practice may be changed as a consequence of reflection, which leads to a modification of declarative knowledge; this new declarative knowledge, in turn, enables planning and setting goals for new practices. Through repetition, the new practice may lead to the development of non-declarative knowledge (skills and habits). While this list of sub-processes may not be complete, we suggest that these processes contribute to the continuous variation of knowledge and practice within organizations; and that our integrative perspective offers starting points for a more refined understanding of the sub-processes involved in (intentional) organizational unlearning.

In addition to offering theoretical advancements, the distinction between declarative and non-declarative knowledge may support a better understanding of practical problems related to organizational learning and change. For example, Blackman et al. (2013) have analyzed the mechanisms underlying the introduction of corporate social responsibility (CSR) measures in organizations. In line with our co-evolution perspective, they have suggested that individual mental models co-evolve through accommodation and assimilation processes triggered by cognitive dissonance. They have also identified a need for unlearning and stated that individual habits may need to be changed for corporate social behavior to be implemented. Such an analysis could benefit from a more precise distinction between declarative and non-declarative forms of knowledge and from our analysis of the interplay between different types of knowledge and practice within organizations.

A limitation of this article is the strict focus on cognitive and communication processes. While we do acknowledge the importance of physical and emotional aspects of individual and collective learning processes (Elkjaer, 2004), these have not been the focus of our attention. What we have also not included in our analysis is the role of psychological predispositions that have been suggested to mediate communication processes, such as attitudes, emotional states, or personality traits (Craig, 1999; Vince, 2001; Shipton and Sillince, 2012), as well as cognitive style (Hayes and Allinson, 1998). In addition, interpersonal relations and mechanisms such as trust or perceived authenticity may have an impact on the verbal or non-verbal communication of knowledge within organizations (Mazutis and Slawinski, 2008; Obembe, 2013). Future work should analyze the role of emotions, intrapersonal predispositions, and interpersonal relations in the co-evolution of both declarative and non-declarative types of knowledge and their relationship with organizational practice.

To conclude, organizational learning can be seen as a complex, recursive process. Practice is based on knowledge, the development of which is always a by-product of practice. In a continuous cycle, modifications in behavior enable new individual and collective experiences that, in turn, may lead to the creation of new declarative and non-declarative knowledge. While it is a challenge to grasp the many complex underlying mechanisms, the conceptual analysis set forth in this article provides a number of starting points for further theoretical and empirical studies devoted to meeting this challenge. Comprehension of the underlying processes will improve the understanding of many knowledge-based phenomena that are the foundations of organizational learning.

## Conflict of Interest Statement

The authors declare that the research was conducted in the absence of any commercial or financial relationships that could be construed as a potential conflict of interest.
